# Submuscular plating vs. elastic stable intramedullary nailing for diaphyseal femur fractures in children: a systematic review and meta-analysis

**DOI:** 10.3389/fped.2023.1256630

**Published:** 2023-11-08

**Authors:** Donghui Li, Xiangyue Wang, Jialing Lu, Mingfeng Xue

**Affiliations:** ^1^ Department of Pediatric Orthopedic, The Second Affiliated Hospital of Jiaxing University, Jiaxing, China; ^2^ Department of Radiation, The Second Affiliated Hospital of Jiaxing University, Jiaxing, China

**Keywords:** pediatric femoral shaft fracture, submuscular plating, elastic stable intramedullary nailing, systematic review, meta-analysis

## Abstract

**Objectives:**

This review evaluates the safety and efficacy of submuscular plating (SMP) vs. elastic stable intramedullary nailing (ESIN) in the treatment of pediatric femur shaft fracture.

**Method:**

Studies comparing the efficacy and safety of SMP and ESIN in pediatric shaft fracture were retrieved from five databases (PubMed, Embase, Cochrane, OVID, and Web of Science) from inception to March 2023 using a systematic literature search strategy. A total of 13 outcome measures, such as perioperative parameters, clinical outcomes, and radiographic results, were included in the meta-analysis.

**Results:**

Eight eligible studies involving 491 patients were included in the narrative synthesis. There were no significant differences in baseline characteristics between the two groups. Meta-analysis showed reduced radiation time (RT), soft tissue irritation and angular deformation in the SMP group than in the ESIN group. However, the SMP group had greater estimated blood loss (EBL) than the ESIN group. The duration of surgery, length of hospital stay (LOS), implant removal, complications requiring surgery, Flynn score, incidence of infection, fracture healing time, and limb length discrepancy (LLD) were similar between the two groups. Only one study reported higher incidences of fracture nonunion or delayed healing in the ESIN group.

**Conclusion:**

SMP is an effective and safe intervention superior to ESIN in reducing soft tissue irritation, angular deformation and radiation time. Given the presence of potential bias and heterogeneity, surgeons should select the treatment that would provide the best outcomes for EBL, LOS, operation time, and bone nonunion or delayed healing based on their experience.

**Systematic Review Registration:**

https://www.crd.york.ac.uk/prospero/display_record.php?ID=CRD42023404118, Identifier PROSPERO (CRD42021228512).

## Introduction

Pediatric femoral fractures are the second most common diaphyseal fracture and the most common pediatric orthopedic injury requiring hospitalization (1), representing about 2% of all pediatric fractures (2). Despite the consensus on the surgical treatment for patients over five years old (3), the selection of fixation method in skeletally immature patients remains controversial (4, 5).

An optimal surgical strategy for pediatric femoral shaft fracture depends on the age and weight of the child, characteristics of the fracture, and accompanying damage (4). In addition, an effective fixation approach should provide adequate primary stability to permit early mobilization, even early weight bearing, maintain fracture biology, minimize scaring and tissue irritation, prevent blood transfusion and serious complications, as well as reduce operation time and radiation exposure (6). The main options for surgical fixation include external fixation, open or submuscular plating fixation, rigid intramedullary nailing, and ESIN (7). External fixation is frequently associated with complications, and open reduction and internal fixation have increased risks of blood transfusion and impaired blood supply to the fracture site. Furthermore, several studies have reported avascular necrosis in rigid intramedullary nailing (8–10). On the other hand, ESIN and SMP have become the most popular fixation approaches for pediatric femoral shaft fractures in recent years (11, 12). ESIN has always been the preferred method of fixation for length stable femoral shaft fracture (13), and has recently demonstrated positive clinical outcomes with low incidences of complications in unstable femoral shaft fracture (14). SMP is an alternative option for length-unstable femoral fractures due to minimal invasion and soft tissue irritation, reliable initial stability that allows for early range of motion (ROM), and satisfactory healing rate, which increased its applicability over ESIN (15, 16).

Both approaches have been recommended in guidelines, but high-level evidence on their efficacy and safety is lacking (3, 15). As a result, neither of these techniques is considered the gold standard, and the selection of either approach remains highly debated (17). A few meta-analyses have compared ESIN with other procedures (18, 19) but not with SMP. Therefore, this study was conducted to evaluate the clinical efficacy, radiology outcomes and safety of SMP vs. ESIN in treating pediatric femoral fractures.

## Methods

### Search strategy

This study was performed in accordance with the Preferred Reporting Items for Systematic Reviews and Meta-Analysis (PRISMA) 2020 statement (20) and was registered in PROSPERO (CRD42021228512). Studies comparing the efficacy and/or safety of SMP and ESIN in pediatric femoral shaft fracture and published in English were systematically searched in PubMed, Embase, The Cochrane Library, and Web of Science from inception to March 2023 using the following terms: “Femoral Fractures”, “Femoral Fracture”, “Fracture, Femoral”, “Fractures, Femoral”, “elastic stable intramedullary nailing”, “elastic stable intramedullary nails”, “elastic stable intramedullary nail”, “elastic nailing”, “elastic nail”, “elastic nails”, “Flexible Intramedullary Nail”, “Flexible Intramedullary Nails”, “flexible intramedullary nailing”, “flexible nails”, “flexible nail”, “flexible nailing”, “Submuscular plating”, “bridge plating”, and “Submuscular bridge plating”. The detailed search strategy is shown in Supplementary Text S1. The references of all eligible studies were also manually searched. All included studies were evaluated independently by two investigators, and any disagreement was resolved by consensus.

### Eligibility criteria

The inclusion criteria were as follows: (1) Randomized-controlled trial (RCT), cohort study, or case-control study; (2) Conducted in skeletally immature children aged five years or older; (3) Study included closed or Gustilo I femoral shaft fractures without other injuries that require concomitant treatment; (4) At least one perioperative measure, including operation time, EBL, LOS, incidence of complication, and radiation expose time; (5) Sufficient data for calculating odds ratio (OR) or weighted mean difference (WMD).

The exclusion criteria were: (1) Reviews, letters, editorial comments, case reports, conference abstracts, adult studies, non-clinical studies, unpublished articles, and non-English articles; (2) Outcome measures cannot be extracted completely; (3) Fewer than 20 cases in two groups (SMP and ESIN); and (4) Patients on whom more than one internal fixation method has been applied.

Postoperative plaster assisted fixation was not excluded from our analysis since it is a routine procedure performed in pediatric fracture surgery and should not have an impact on the study results.

### Data extraction

Data were extracted independently by two investigators, and any discrepancy was resolved by discussion with a third investigator. Extracted data included study characteristics (first author, publication year, study period, country of study, study type, number of cases), demographics (patient age, patient weight, gender, fracture stability), perioperative parameters (duration of surgery, EBL, RT and LOS), clinical outcomes (soft tissue irritation, implant removal, complications requiring surgery, Flynn score, and infections), and radiographic outcomes (fracture healing time, angular deformation, LLD, and fracture non-union or delayed healing). Continuous variables were expressed as mean ± standard deviation using a validated mathematical method (21, 22).

### Quality assessment

Quality assessment was performed using the Cochrane Collaboration's risk of bias assessment (RoB) tool for RCTs and the Newcastle–Ottawa Scale (NOS) for retrospective and prospective cohort studies. Studies with a score of 7–9 were regarded as high-quality. Discrepancies in quality assessment results were resolved through discussion between the two independent investigators.

### Statistical analysis

Evidence synthesis was completed using Review Manager 5.3 (Cochrane Collaboration, Oxford, UK). Continuous and categorical variables were compared between groups using WMD and OR with confidential intervals (CIs), respectively. Heterogeneity among studies was assessed by the chi-squared (χ^2^) test (Cochran's Q) and inconsistency index (*I*^2^) (23). When *I*^2 ^> 50%, a random-effects model was used for data analysis; otherwise, a fixed-effects model was used. Furthermore, sensitivity analyses were performed for outcomes with significant heterogeneity to evaluate the effect of the included studies. Publication bias was evaluated using funnel plots in Review Manager 5.3 (Cochrane Collaboration, Oxford, UK).

## Results

### Literature search and study characteristics

**Figure 1:**
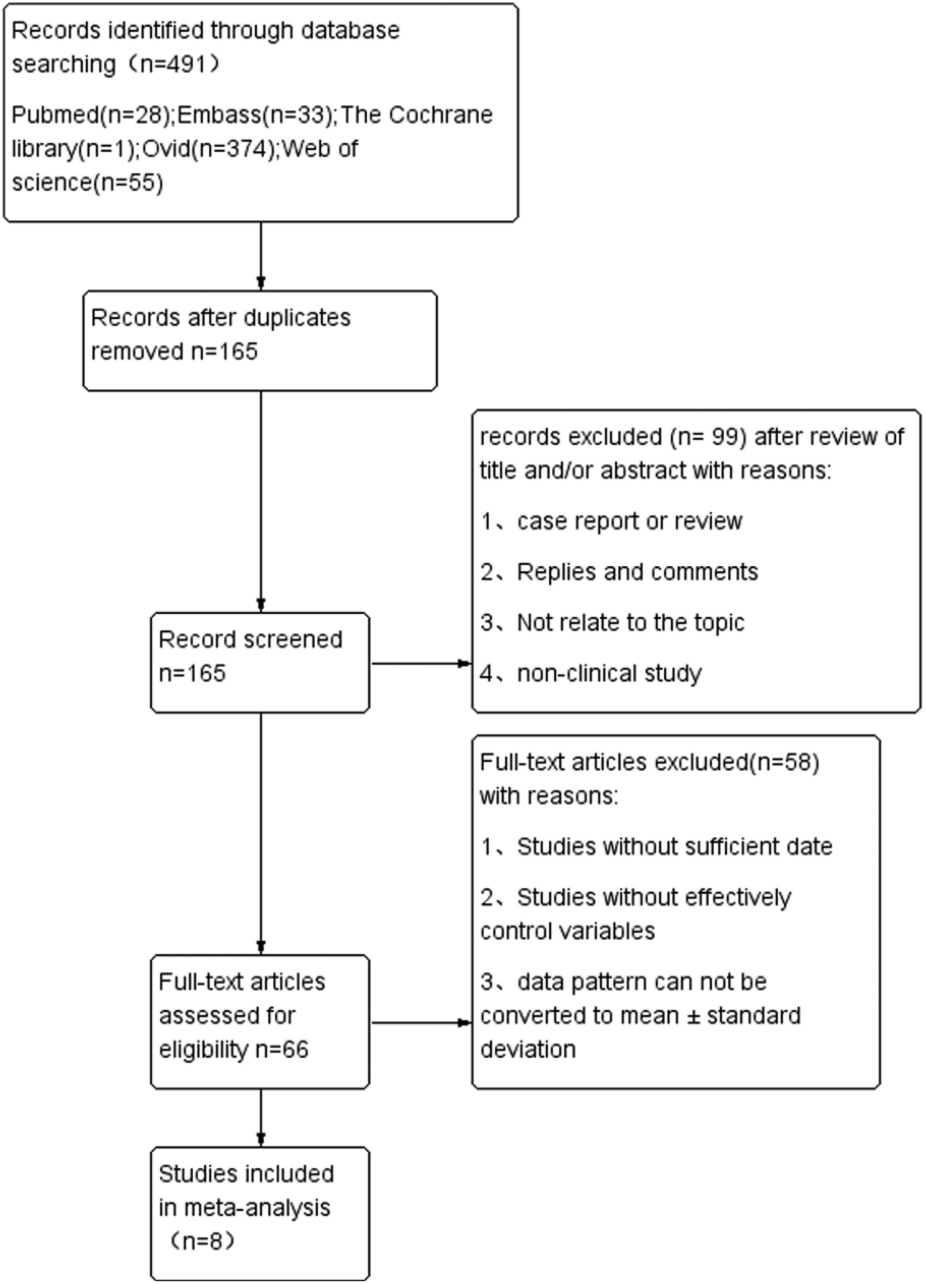
Flowchart of study screening.

**Figure 2:**
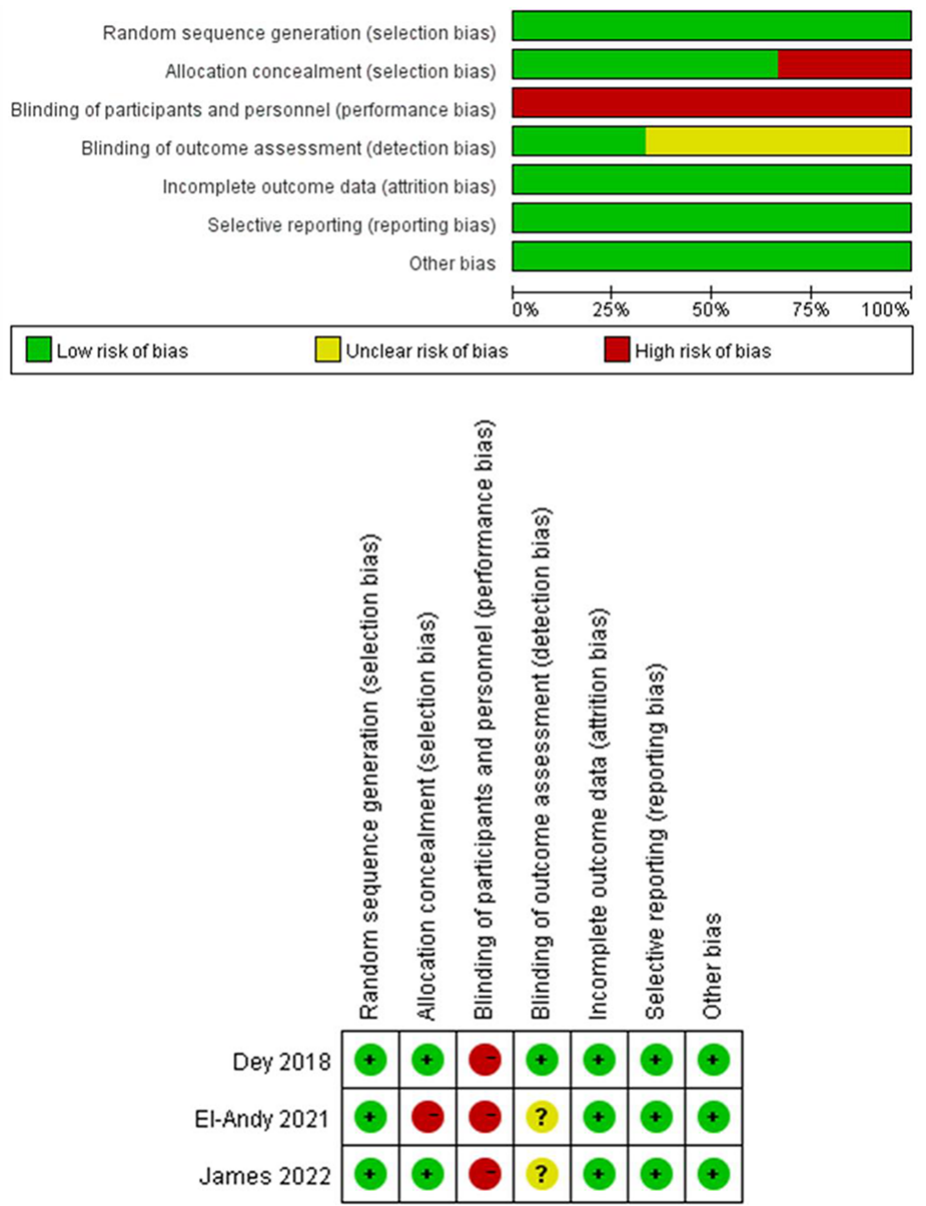
Publication bias in RCTs summary of the risk of bias across the 3 included RCTs; judgements regarding each risk of bias item for each included study.

We initially identified 491 relevant publications from Pubmed (*n* = 28), Cochrane Library (*n* = 1), Embase (*n* = 33), OVID (*n* = 374), and Web of Science (*n* = 55). The study selection process is shown in Figure 1. After removing duplicates, the title and abstract of 165 articles were screened, and the full texts of 66 articles were assessed for eligibility. Finally, 8 studies involving 491 patients (216 in SMP group, 275 in ESIN group) were included in the meta-analysis (12, 24–30), including 2 prospective cohort studies (25, 27), 3 retrospective cohort studies (24, 28, 29), and 3 RCTs (12, 26, 30). The characteristics and quality scores of each included study are summarized in Table 1. All the included prospective and retrospective cohort studies were identified as high quality according to NOS (Supplementary Table S1). In addition, the RCTs were also of good quality with a low risk of bias according to the RoB assessment (Figure 2).

**Table 1:** Baseline characteristics of include studies and methodological assessment.

Authors	Study period	Country	Study design	Patients (*n*)	Median follow-up (months)	Quality score
				SMP/ESIN		
Chen et al. (25)	2005.1–2017.6	USA	Prospective cohort	30/28	22	8
Milligan et al. (28)	2009.4–2017.4	UK	Retrospective cohort	14/14	63.6	8
Li et al. (27)	2008.1–2018.1	CH	Prospective cohort	45/77	24	9
Sutphen et al. (24)	2001.1–2014.10	USA	Retrospective cohort	35/61	48	8
Yigit et al. (29)	–	Turkey	Retrospective cohort	28/32	29.8	9
Dey et al. (26)	2011.3–2015.4	India	RCT	19/18	26.2	–
El-Adly et al. (30)	2018–2020	India	RCT	25/25	12	–
James et al. (12)	2013.1–2016.6	India	RCT	20/20	24	–

SMP, submuscular locked plate; ESIN, elastic stable intramedullary nailing; RCT, randomized controlled trial.

### Demographics

No significant differences were noted in patient age (WMD: −0.16; 95% CI: −0.73, 0.41; *P* = 0.58), gender (WMD: −0.16; 95% CI: −0.73, 0.41; *P* = 0.58), weight (WMD: −0.16; 95% CI: −0.73, 0.41; *P* = 0.58) and fracture stability (WMD: −0.16; 95% CI: −0.73, 0.41; *P* = 0.58) (Table 2).

**Table 2:** Demographics and clinical characteristics of included studies.

Outcomes	Studies	No. of patients	WMD or OR	95% CI	*p*-value	Heterogeneity			
		SMP/ESIN				Chi^2^	df	*p*-value	*I*^2^ (%)
Age (years)	7	181/214	0.10	[−0.74, 0.94]	0.82	36.28	6	<0.00001	83
Gender (male)	8	216/275	1.13	[0.77, 1.68]	0.53	5.52	7	0.60	0
Weight (kg)	4	112/147	1.30	[−0.68, 3.27]	0.20	7.63	3	0.05	61
Unstable fracture	7	202/261	3.00	[0.46, 19.62]	0.25	10.79	2	0.005	81
Duration of surgery	5	137/172	7.07	[−1.01, 15.15]	0.09	67.16	4	<0.00001	94
RT (s)	5	127/156	−17.30	[−29.02, −5.58]	0.004	59.80	4	<0.00001	93
EBL (ml)	5	137/172	27.31	[4.39, 50.24]	0.02	105.58	4	<0.00001	96
LOS (day)	5	132/168	0.62	[−0.10, 1.35]	0.09	180.63	4	<0.00001	98
Soft tissue irritation	7	188/243	0.15	[0.07, 0.31]	<0.00001	1.43	6	0.96	0
Implant remove	6	163/218	1.98	[0.72, 5.43]	0.18	9.52	4	0.05	58
Flynn score (excellent)	5	131/166	1.97	[0.72, 5.39]	0.19	10.16	4	0.04	61
Flynn score (satisfactory and excellent)	5	131/166	1.27	[0.34, 4.69]	0.72	0.50	2	0.78	0
Infection	3	69/66	0.76	[0.21, 2.80]	0.68	0.98	2	0.61	0
Fracture union time (week)	4	92/95	−0.50	[−1.26, 0.26]	0.20	68.99	3	<0.00001	96
Angular deformation	4	125/183	0.40	[0.16, 0.99]	0.05	0.41	3	0.94	0
LLD	8	216/275	0.80	[0.20, 3.22]	0.75	0.95	2	0.62	0
Non-union or delay healing	8	216/275	0.18	[0.01, 3.44]	0.25	–	–	–	–

SMP, submuscular locked plate; ESIN, elastic stable intramedullary nailing; WMD, weighted mean difference; OR, odds ratio; CI, confidence interval; RT, radiation time; EBL: establish blood loss; LOS: length of hospital stay; LLD, limb length discrepancy.

### Perioperative parameters

#### Duration of surgery

**Figure 3:**
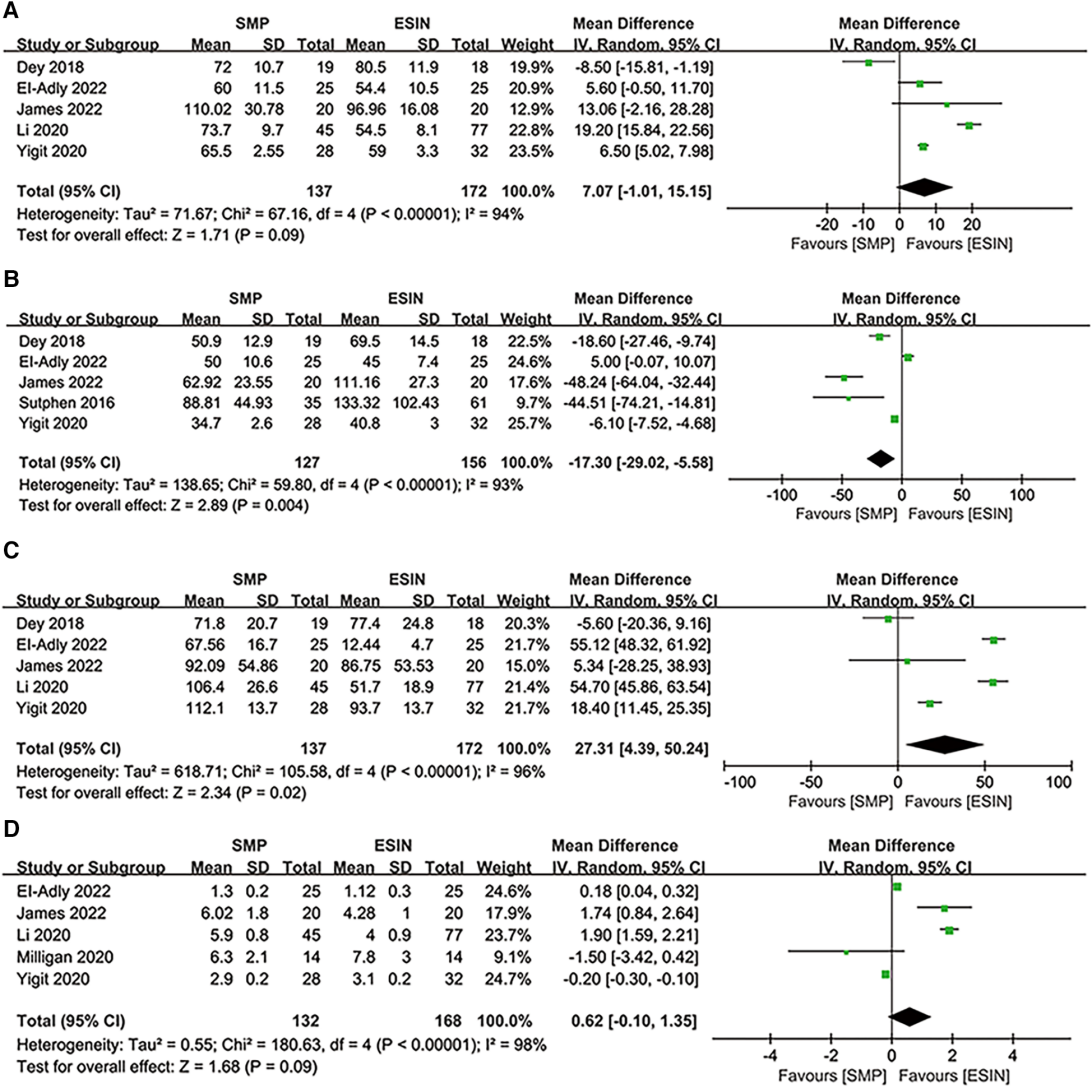
Forest plots of perioperative parameters: (**A**) duration of surgery; (**B**) radiological time; (**C**) estimated blood losing; (**D**) length of stay.

Duration of surgery was reported in 5 studies including 309 patients (137 in SMP and 172 in ESIN) (12, 26, 27, 29, 30). No significant difference was observed in the duration of surgery between the two groups [WMD: 7.07; 95% CI: (−1.01, 15.15); *P* = 0.09], but there was significant heterogeneity among the studies (*I*^2^ = 94%, *P* < 0.00001) (Figure 3A).

#### RT

Five studies involving 283 patients (127 in SMP and 156 in ESIN) reported radiation time (12, 24, 26, 29, 30). The pooled results showed that radiation time was significantly shorter in the SMP group than in the ESIN group [WMD, −17.3; 95% CI: (−29.02, −5.58); *P *= 0.004]. There was significant heterogeneity among the studies (*I*^2 ^= 93%, *P *< 0.00001) (Figure 3B).

#### EBL

Five studies (12, 26, 27, 29, 30) reported EBL. The meta-analysis showed significantly greater EBL in SMP than in ESIN [WMD = 27.31, 95% CI: (4.39, 50.24), *P *= 0.02], with significant heterogeneity among the studies (*I*^2 ^= 96%, *P *< 0.00001) (Figure 3C).

#### LOS

**Figure 4:**
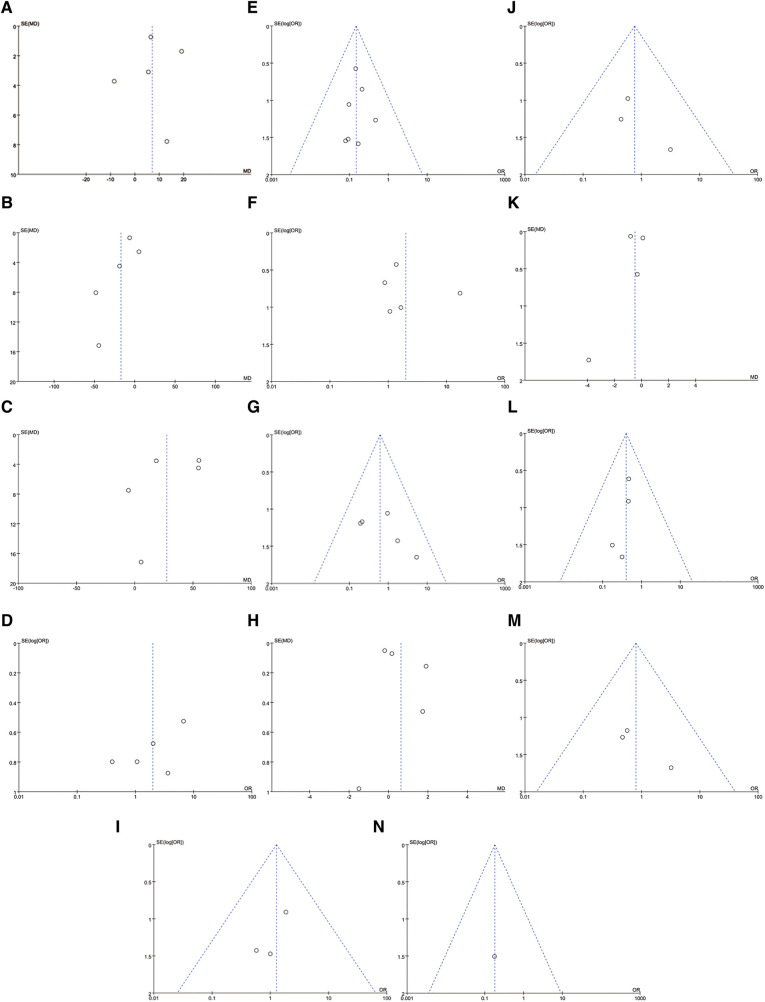
Funnel plots: (**A**) duration of surgery; (**B**) radiological time; (**C**) estimated blood losing; (**D**) length of stay; (**E**) soft tissue irritation; (**F**) implant remove; (**G**) comlication need to surgery; (**H**) flynn score(excellent and satisfactory); (**I**) flynn score(excellent); (**J**) infection; (**K**) fracture union time; (**L**) angular deformation; (**M**) limb length discrepancy; (**N**) non-union or delay union.

Five studies (*n* = 300) discussed LOS (132 in SMP and 168 in ESIN) (12, 27–30). Meta-analysis showed similar LOS between the two groups [WMD: 0.62; 95% CI: (−0.1, 0.35); *P *= 0.09], with significant heterogeneity among studies (*I*^2 ^= 98%, *P *< 0.0001) (Figure 3D). Furthermore, funnel plots revealed a slight publication bias in all of the above four perioperative parameters (Figures 4A–D).

### Clinical outcomes

#### Soft tissue irritation

**Figure 5:**
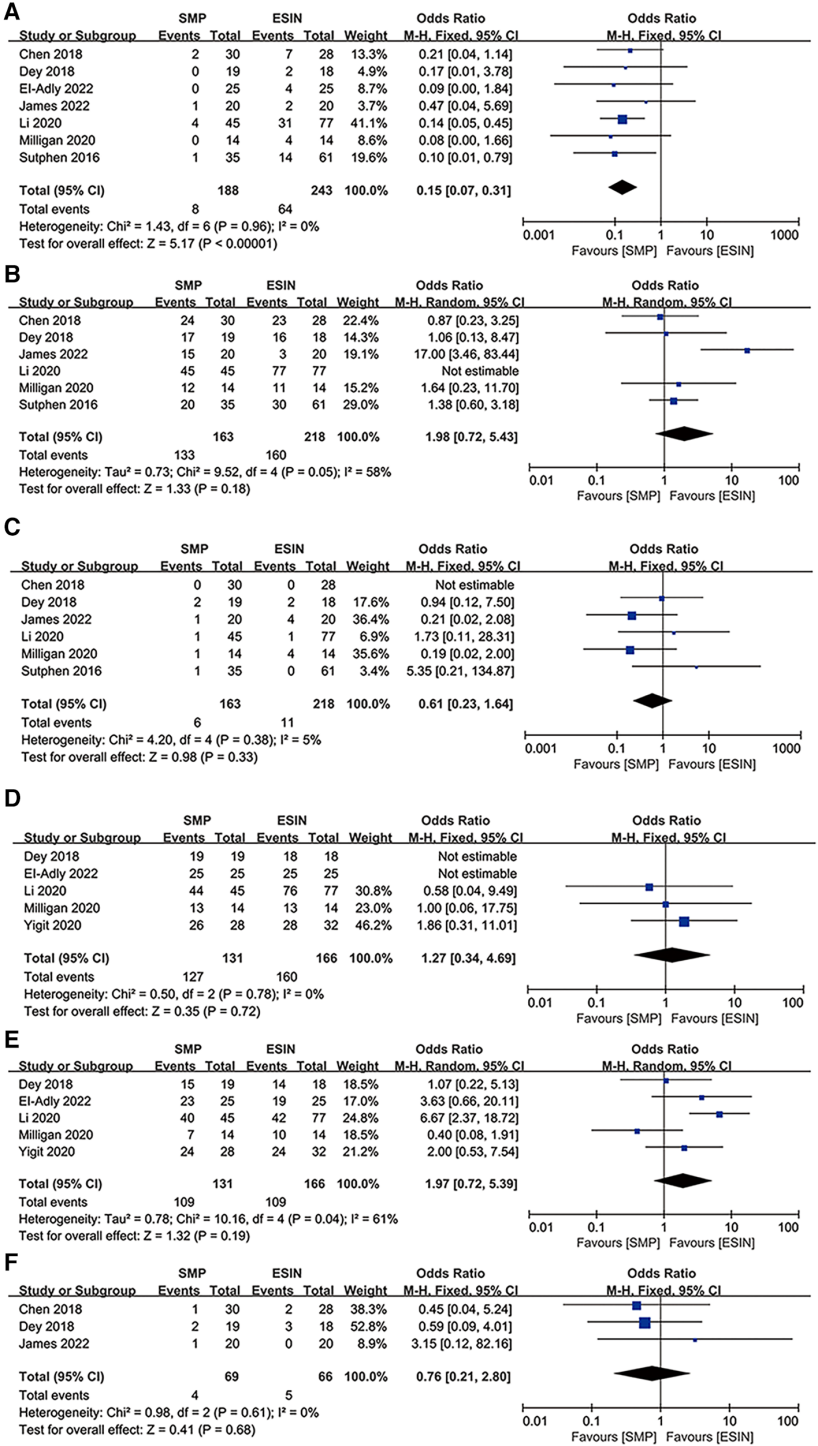
Forest plots of clinical outcomes: (**A**) soft tissue irritation; (**B**) implant remove; (**C**) comlication need to surgery; (**D**) flynn score(excellent and satisfactry); (**E**) flynn score(excellent); (**F**) infection.

Five studies reported soft tissue irritation, encompassing 431 patients (188 in SMP and 243 in ESIN) (12, 24–28, 30). The analysis results indicated significantly lower soft tissue irritation in the SMP group than in the ESIN group [OR: 0.15; 95% CI: (0.01, 0.37), *P *< 0.0001], with no significant heterogeneity among the studies (*I*^2 ^= 0%, *P *= 0.96) (Figure 5A).

#### Implant removal

Implant removal was reported in six studies with a total of 381 patients (12, 24–28), of whom 296 decided to remove their internal fixation devices (44.93% in SMP and 55.07% in ESIN). No significant difference was found in implant removal [OR: 1.98; 95% CI: (0.72, 5.43), *P* = 0.18]. However, there was moderate heterogeneity (*I*^2 ^= 58%, *P *= 0.18) (Figure 5B) and slight publication bias in the studies (Figure 4F).

#### Complications requiring surgery

Although all 8 studies reported postoperative complications, meta-analysis was performed on only 5 studies, involving 17 cases (6 SMP vs. 11 ESIN) of complication in 323 patients (12, 24, 26–28). There was no significant difference in the incidences of complications requiring surgery between the two groups [OR: 0.61; 95% CI: (0.23, 1.64), *P *= 0.33] and no significant heterogeneity among the studies (*I*^2 ^= 5%, *P *= 0.38) (Figure 5C). Reasons for secondary surgery were re-fracture, loss of reduction, deep infection, delayed union or non-union, and excessive deformity.

#### Flynn score

Flynn scores are rarely or poorly reported in most studies, and thus we performed subgroup analyses according to that patients have an Excellent or Satisfactory result or have an Excellent result only. We then further compared the clinical efficacy of the two interventions.

Flynn scores for child femoral shaft frame were pooled from 5 studies (26–30) involving 297 subjects (131 in SMP and 166 in ESIN). Neither the Excellent/Satisfactory [OR: 1.27; 95% CI: (0.34, 4.69), *P *= 0.72] nor Excellent subgroups [OR: 1.97; 95% CI: (0.72, 5.39), *P *= 0.19] showed a significant difference in the number of patients between the two interventions. There was significant heterogeneity in the latter subgroup (*I*^2 ^= 61%, *P *= 0.04) (Figure 5E) but not in the former subgroup (*I*^2 ^= 0%, *P *= 0.78) (Figure 5D). The funnel plot revealed a slight publication bias in Flynn score in the Excellent subgroup but not in the Excellent/Satisfactory subgroup.

#### Infections

Infections were reported in three studies (12, 25, 26), with an overall incidence of less than 7%. Infections were reported in 3 patients by Chen et al. (SMP: 1/30; ESIN: 2/28), 5 patients by Dey et al. (SMP: 2/19; ESIN: 3/18), and 1 patient by James et al. (SMP: 1/20). There was no significant difference in the incidence of infection between the two groups [OR: 0.76; 95% CI: (0.21, 2.80), *P *= 0.68], and there was no significant heterogeneity in the results (*I*^2 ^= 0%, *P *= 0.61) (Figure 5F).

### Radiographic results

#### Fracture healing time

**Figure 6:**
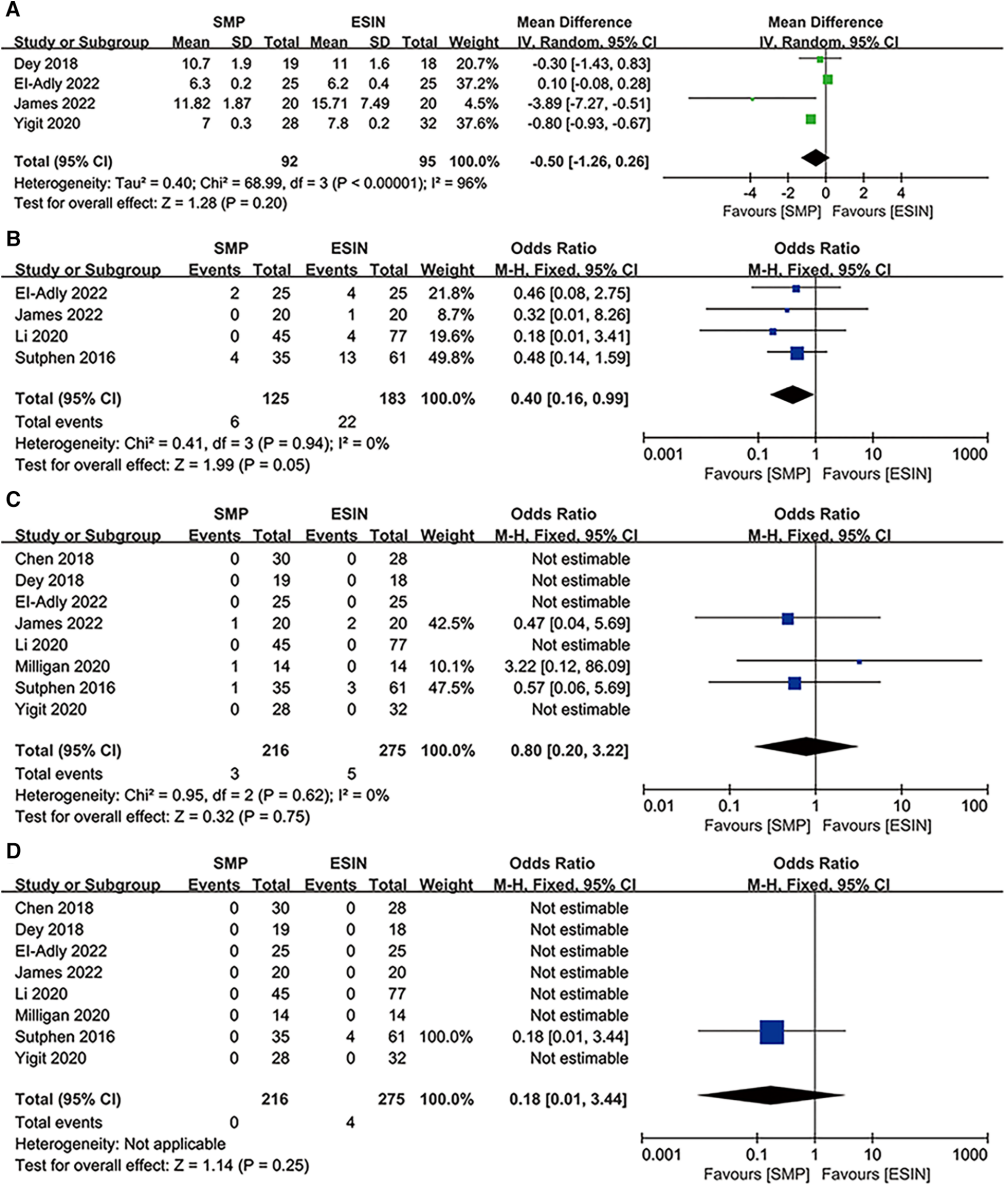
Forest plots of radiographic results: (**A**) fracture union time; (**B**) angular deformation; (**C**) limb length discrepancy; (**D**) non-union or delay union.

Four studies involving 187 patients (92 in SMP and 95 in ESIN) reported fracture healing time (12, 26, 29, 30), which was defined as the first observation of cortical continuity in more than 3 directions on the anteroposterior lateral x-ray. Meta-analysis indicated no significant difference in fracture healing time between the two groups [OR: −0.5; 95% CI: (−1.26, 0.26), *P *= 0.20]. However, there was significant heterogeneity (*I*^2 ^= 96%; *P *< 0.00001) (Figure 6A) and publication bias (Figure 4K).

#### Angular deformation

Four studies involving 308 patients (125 in SMP and 183 in ESIN) (12, 24, 27, 30) discussed angular deformation. The results revealed that the SMP group had a significantly lower angular deformation rate than the ESIN group [OR: 0.4; 95% CI: (0.16, 0.99); *P* = 0.05] (Figure 6B), with no significant heterogeneity (*I*^2^ = 0%, *P* = 0.94) (Figure 4L).

#### LLD

The incidence of complication was reported in 8 studies with 491 patients (216 in SMP and 275 in ESIN) (12, 24–30). Meta-analysis indicated no significant difference in the incidence of complication between the two groups [OR: 0.8; 95% CI: (0.20, 3.22); *P* = 0.75] (Figure 6C), with no heterogeneity (*I*^2 ^= 0%, *P *= 0.75) (Figure 4M).

#### Nonunion or delayed healing

Only Sutphen et al. reported 4 cases of nonunion or delayed healing, all of which were observed in the ESIN group (Figure 6D).

### Sensitive analysis

**Figure 7:**
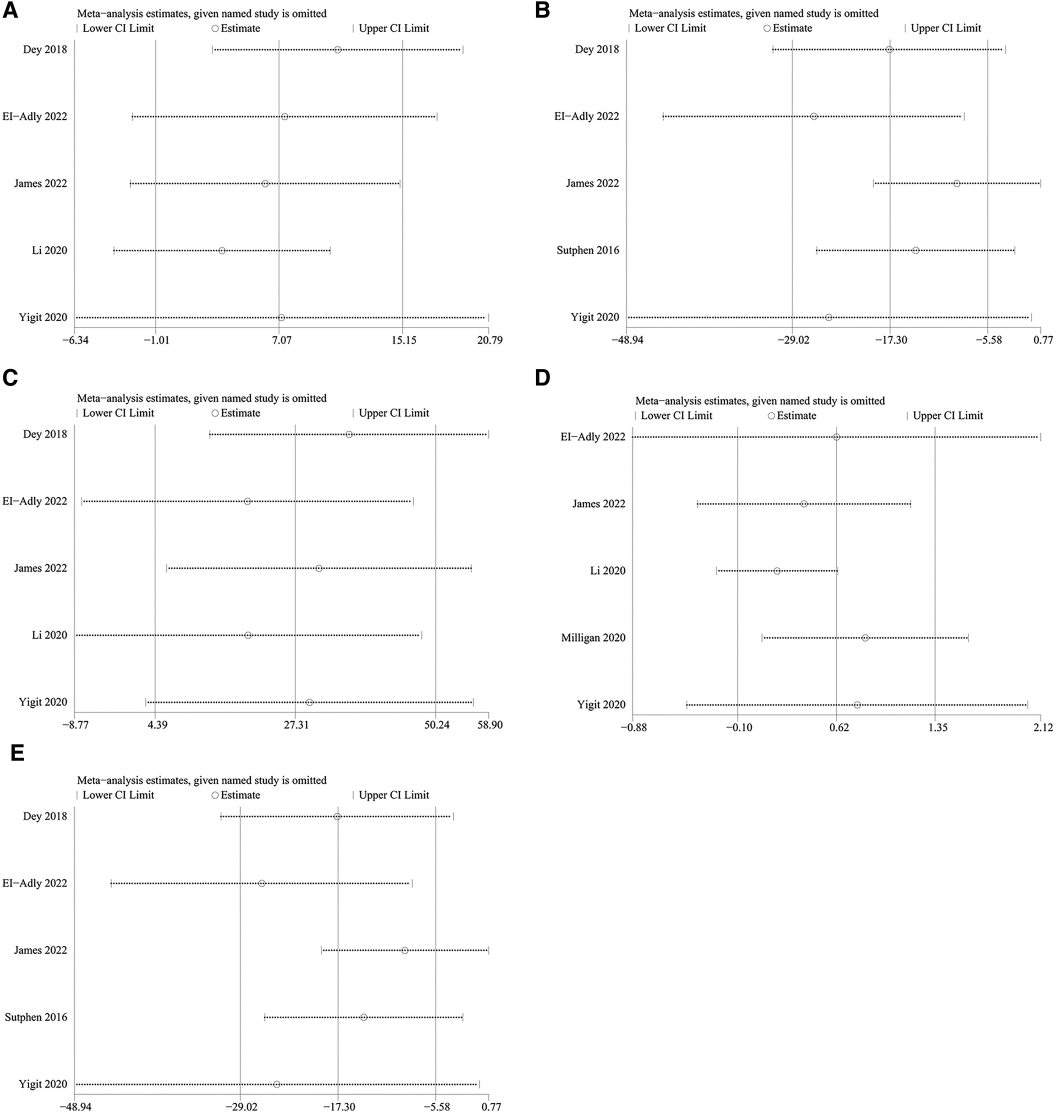
Sensitively analysis of: (**A**) duration of surgery; (**B**) radiological time; (**C**) estimated blood losing; (**D**) length of stay; (**E**) fracture union time.

To evaluate the influence of each individual study on the pooled WMD, we performed one-way sensitivity analyses on duration of surgery, RT, EBL, LOS, and fracture healing time (Figure 7). The pooled WMD for RT and fracture healing time remained unchanged after excluding each individual study. However, with the presence of heterogeneity for LOS and duration of surgery after the exclusion of Dey et al. for the former and Milligan et al. for the latter, the statistical significance of the results has changed [LOS: *P *= 0.009, WMD: 10.92, 95% CI: (0.07, 1.59); Duration of surgery: *P *= 0.009, WMD = 10.92, 95% CI: (2.71, 19.12)]. The heterogeneity in EBL also decreased (*I*^2 ^= 77%, *P *= 0.45) when the two studies (27, 30) were excluded, and the significant difference in EBL disappeared [WMD: 7.16, 95% CI: (−11.50, 25.81), *P *= 0.45]. Collectively, these results indicate that the above studies were the primary sources of heterogeneity for these parameters.

## Discussion

Children over the age of 5 with femoral shaft fractures have been previously shown to benefit from surgical treatment (31). However, there is currently no clear consensus on the optimal method of fixation. Commonly used fixation approaches include external fixation, rigid antegrade intramedullary nailing, common plate, SMP and ESIN. Among them, ESIN and SMP are the most widely used approaches recommended by guidelines (15). However, given potential adverse events, further research into the best treatment is warranted.

In this meta-analysis, we extracted 13 parameters from the eligible studies. Four parameters were significantly different between the two groups, while eight parameters were not. Bone nonunion or delayed healing was only reported by one study, and both occurred in the ESIN group. For perioperative parameters, the radiation exposure time was shorter in the SMP group than in the ESIN group, possibly because multiple fluoroscopies are required during ESIN fixation to achieve a more precise reduction. Studies have (32, 33) hinted that surgeons should consider the potential damages of this procedure when planning the surgical strategy. In addition, we found that the SMP group had significantly greater EBL than the ESIN group. Though, the additional bleeding in the children did not cause shock or require blood transfusions. Therefore, EBL does not have a major influence on the selection of interventions.

The ESIN group had a higher incidence of soft tissue irritation than the SMP group. These irritation symptoms partially led to early removal of internal fixation devices, wound complications, and decreased patient satisfaction. Other studies reported an equally high incidence of soft tissue irritation in ESIN (34, 35). Moreover, the ESIN group exhibited higher degrees of angularity during follow-up. It was reported that the weight of patients may influence the incidence of angular deformities after ESIN (36, 37). Therefore, the child's weight should be taken into account during ESIN. In unstable fractures, ESIN was found to be inadequate for controlling rotations whereas SMP provided good stability (38, 39). These evidence indirectly supports the reliability of this conclusion.

ESIN is associated with several surgical complications, including angular deformities, soft tissue irritation, and LLD (40), which are more common in length-unstable femoral fractures and fractures that involve the proximal or distal third of the femur (13). As a result, many researchers have shifted to SMP for pediatric femoral shaft fractures and achieved positive outcomes in children with length unstable fractures or greater body weight or fractures involving one-third of the distal and proximal ends. Nonetheless, no meta-analysis of the efficacy and safety of SMP and ESIN was conducted before. Here, we pooled the data from 8 studies published before 2023 and found that SMP results in a lower incidence of angular deformities. Body weight, fracture stability, and the thickness of ESIN may impact the results. Across the four studies reporting angular deformation, no statistical differences were found in baseline fracture stability [OR: 1.67; 95% CI: (0.08, 36.53); *P* = 0.74] between SMP and ESIN groups. Our results possibly indicate that SMP can provide more fixation stability. Conversely, the four study does not report the thickness of ESIN and only two of them provided body weight, so we cannot ignore the impact of ESIN thickness and body weight on the incidence of angular deformity. Due to their low incidence rates, delayed fracture healing and nonunion were reported in only one study, and all cases were observed in the ESIN group.

There were no significant differences in the incidences of postoperative infections, patient satisfaction, and postoperative complications requiring surgery between the two groups. While SMP involves a larger incision and hence greater soft tissue damage than ESIN, there was no increased risks of soft tissue complications such as exudation, infection, and wound dehiscence. Although the ESIN group has a higher incidence of angular deformities, our meta-analysis did not indicate a higher risk of LLD, which is contrary to the results of some case-control studies (34, 40, 41). In addition, while ESIN was associated with longer radiation exposure time, it did not affect fracture healing time, which may be attributed to the presence of both closed reduction and elastic fixation. Notably, the pooled results for both parameters were highly heterogeneous, and sensitivity analysis did not identify the source of heterogeneity. Hence, these findings should be interpreted with caution. The removal of studies did not abrogate the heterogeneity in LOS and surgical time but altered the statistical significance of the differences between the two groups. The new WMD indicated that SMP results in longer surgical time and LOS than ESIN. Furthermore, sensitivity analysis reduced the heterogeneity in EBL and abrogated the statistical significance between the two groups. We speculate that the stability of the results may be influenced by two studies (27, 30), and EBL is associated with the surgical skills and experience of the surgeon.

However, there are still several limitations to our study. First, the number of included studies was small and not all of them were RCTs. Due to the lack of high-quality research in this area, further RCTs are warranted to provide more reliable clinical results. Second, there is still high heterogeneity in LOS, surgical time, RT, and fracture healing time. Although we have conducted sensitivity analysis, the sources of heterogeneity could not be eliminated. Therefore, these results should be interpreted with caution. Furthermore, due to limited studies, effective conclusions cannot be drawn on the incidence of bone nonunion and delayed healing. Last, the removal of internal fixation devices was recommended in both groups, which may affect the reliability of the results. The operation time for their removal and postoperative complications are meaningful research topics, but due to insufficient data from the literature, these parameters could not be examined in this study.

## Conclusion

SMP is an effective and safe intervention superior to ESIN in reducing soft tissue irritation, angular deformation, and radiation time. However, Flynn score, incidence of infection, fracture healing time, and LLD are similar between the two approaches. Given the presence of potential bias and heterogeneity, surgeons should select the treatment that would provide the best outcomes for EBL, LOS, operation time, and bone nonunion or delayed healing based on their experience.

## Data Availability

The original contributions presented in the study are included in the article/**Supplementary Material**, further inquiries can be directed to the corresponding author.
