# Supplementary material for: Submuscular plating vs. elastic stable intramedullary nailing for diaphyseal femur fractures in children: a systematic review and meta-analysis
Source: Front Pediatr. 2023 Nov 8;11:1256630. doi: 10.3389/fped.2023.1256630 (PMC10663353; doi:10.3389/fped.2023.1256630)
Supplement: Supplementary file 1 [file Datasheet1.pdf]

Supplementary Text S1 Search strategy

(("Submuscular"[All Fields] AND ("bone plates"[MeSH Terms] OR ("bone"[All Fields] AND "plates"[All Fields]) OR "bone plates"[All Fields] OR "plate"[All Fields] OR "plate s"[All Fields] OR "plated"[All Fields] OR "plates"[All Fields] OR "plating"[All Fields] OR "platings"[All Fields])) OR (("bridge"[All Fields] OR "bridged"[All Fields] OR "bridges"[All Fields] OR "bridging"[All Fields] OR "bridgings"[All Fields]) AND ("bone plates"[MeSH Terms] OR ("bone"[All Fields] AND "plates"[All Fields]) OR "bone plates"[All Fields] OR "plate"[All Fields] OR "plate s"[All Fields] OR "plated"[All Fields] OR "plates"[All Fields] OR "plating"[All Fields] OR "platings"[All Fields])) OR ("Submuscular"[All Fields] AND ("bridge"[All Fields] OR "bridged"[All Fields] OR "bridges"[All Fields] OR "bridging"[All Fields] OR "bridgings"[All Fields]) AND ("bone plates"[MeSH Terms] OR ("bone"[All Fields] AND "plates"[All Fields]) OR "bone plates"[All Fields] OR "plate"[All Fields] OR "plate s"[All Fields] OR "plated"[All Fields] OR "plates"[All Fields] OR "plating"[All Fields] OR "platings"[All Fields]))) AND ("elastic stable intramedullary nailing"[Title/Abstract] OR "elastic stable intramedullary nails"[Title/Abstract] OR "elastic stable intramedullary nail"[Title/Abstract] OR "elastic nailing"[Title/Abstract] OR "elastic nail"[Title/Abstract] OR "elastic nails"[Title/Abstract] OR "flexible intramedullary nail"[Title/Abstract] OR "flexible intramedullary nails"[Title/Abstract] OR "flexible intramedullary nailing"[Title/Abstract] OR "flexible nails"[Title/Abstract] OR "flexible nail"[Title/Abstract] OR "flexible nailing"[Title/Abstract]) AND ("femoral fracture"[Title/Abstract] OR "fracture femoral"[Title/Abstract] OR "fractures femoral"[Title/Abstract] OR "Femoral Fractures"[MeSH Terms])

Supplementary Table S1 Quality evaluation of the eligible studies with Newcastle–Ottawa scale.

Study	Selection				Comparability		Outcome		
	Representative-ness	Selection of non-exposed	Ascertainment of exposure	Outcome not present at start	Comparability on most important factors	Comparability on other risk factors	Assessment of outcome	Long enough follow-up (median≥1 year)	Adequacy (completeness) of follow-up
Chen et al.	*	*	*	*	-	*	*	*	*
Li et al.	*	*	*	*	*	*	*	*	*
Milligan et al.	*	*	*	*	-	*	*	*	*
Sutphen et al.	*	*	*	*	-	*	*	*	*
Yigit et al.	*	*	*	*	*	*	*	*	*

*indicates criterion met; - indicates significant of criterion not met.
